# Computational refocusing of Jones matrix polarization-sensitive optical coherence tomography and investigation of defocus-induced polarization artifacts

**DOI:** 10.1364/BOE.454975

**Published:** 2022-04-22

**Authors:** Lida Zhu, Shuichi Makita, Daisuke Oida, Arata Miyazawa, Kensuke Oikawa, Pradipta Mukherjee, Antonia Lichtenegger, Martin Distel, Yoshiaki Yasuno

**Affiliations:** 1 Computational Optics Group, University of Tsukuba, Tsukuba, Ibaraki, Japan; 2 Sky technology Inc., Tsukuba, Ibaraki, Japan; 3 Center for Medical Physics and Biomedical Engineering, Medical University of Vienna, Vienna, Austria; 4 Innovative Cancer Models, St. Anna Children’s Cancer Research Institute, Vienna, Austria

## Abstract

Here we demonstrate a long-depth-of-focus imaging method using polarization sensitive optical coherence tomography (PS-OCT). This method involves a combination of Fresnel-diffraction-model-based phase sensitive computational refocusing and Jones-matrix based PS-OCT (JM-OCT). JM-OCT measures four complex OCT images corresponding to four polarization channels. These OCT images are computationally refocused as preserving the mutual phase consistency. This method is validated using a static phantom, postmortem zebrafish, and *ex vivo* porcine muscle samples. All the samples demonstrated successful computationally-refocused birefringence and degree-of-polarization-uniformity (DOPU) images. We found that defocusing induces polarization artifacts, i.e., incorrectly high birefringence values and low DOPU values, which are substantially mitigated by computational refocusing.

## 1. Introduction

Cultured *in vitro* tissues are widely used in basic medicine and drug development. Recent progress in cultivation techniques has enabled thick and functionally-shaped tissue cultures such as spheroids and organoids [1–4]. Such tissue cultures are not only collections of cells, but can also mimic tissue micro-environments such as the extracellular matrix, making them a promising tool for basic medical and pharmaceutical research. However, such thick tissue cultures present three requirements for their imaging. The first requirement is high resolution of a few micrometers. Second, an imaging depth (image penetration depth) of a few millimeters is required. This requirement becomes more important as culturing techniques improve and the tissue size increases. Third, intrinsic molecular contrast is desirable, especially for collagen and fibrous tissues, which are the main components of the extracellular matrix [5].

Optical coherence tomography (OCT) based microscope, or optical coherence microscope (OCM), has recently attracted the attention of researchers for using as a high-penetration three-dimensional imaging tool for cultured *in vitro* tissue and *ex vivo* tissues. It has resolution down to a few micrometers and imaging penetration of a few millimeters, and has been successfully applied to imaging of *ex vivo* retina [6], corneal graft [7], brain cancer [8], and *in vitro* tumor spheroids [9].

However, conventional OCT and OCM visualize only the scattering properties of tissues, and the molecular contrast provided is therefore weak. Several extensions of OCT have been explored to overcome this limitation. For example, visible-light OCT [10] can measure the absorption spectrum of the sample through the OCT probe beam. And oxygen saturation of blood can be measured through the absorption [11–14]. Namely, visible-light OCT has sensitivity to oxy- and deoxy-hemoglobin. Photothermal OCT can create contrast for a specific molecule by illuminating the sample with excitation light of a specific wavelength, making the light absorbed by the molecule and measuring the optical-path-length alteration caused by the absorption by the molecule [15,16]. Hence, photothermal OCT facilitates detection of a specific molecule through its absorption spectrum.

Polarization sensitive OCT (PS-OCT) is another technique. An example application is measurement of degree-of-polarization uniformity (DOPU) [17] by PS-OCT, which is known to be sensitive to melanin. Tissue birefringence, which is another polarization property measurable by PS-OCT, is mainly caused by fibrous structure within tissue, and birefringence imaging can be used to detect fibrous tissue and collagen [18–20]. PS-OCT potentially fulfills the three requirements of high resolution, high image penetration, and collagen sensitivity.

However, both OCT and PS-OCT are suffered from the trade-off between the lateral resolution and the depth-of-focus (DOF). Namely, high lateral resolution and long DOF cannot be simultaneously achieved. And it limits the one-shot imaging depth achieved by a single measurement of OCT. For non-polarization-sensitive OCT, both hardware- and software-based methods have been demonstrated to overcome this trade-off. The hardware methods include mechanical focal shifting [21] and Bessel-beam-based extended focus [22]. Note that the former extends the total imaging depth but cannot extend the one-shot imaging depth. The software methods include Fresnel-diffraction-model based computational refocusing [23], interferometric synthetic aperture microscopy (ISAM) [24], and computational/digital adaptive optics [25–28]. Hardware-software hybrid methods, such as use of a depth-encoded aperture [29], have also been demonstrated.

Extended depth-of-focus PS-OCT has also been reported. Kwon *et al.* demonstrated PS-OCT with dark-field extended-focus probe optics [30]. However, although their method provides high-penetration polarization-sensitive imaging without elaborate signal processing, it requires complex probe optics. South *et al.* demonstrated a software-based focus extension for PS-OCT [31]. In their method, they combined ISAM and circularly-polarized light based PS-OCT (CPL-PSOCT) [32], and demonstrated cumulative phase retardation imaging with computational refocusing. Similarly, Wang *et al.* demonstrated the combination of computational adaptive optics (CAO) with the CPL-PSOCT [33]. CPL-PSOCT is particularly suitable for computational refocusing because it uses only the intensity information to compute the cumulative phase retardation. And hence the phase retardation computation is less prone to phase-sensitive pre-processing including computational refocusing. However, this non-phase-sensitive method is incompatible with birefringence imaging, i.e., local phase retardation imaging. To measure birefringence by CPL-PSOCT, phase information must be used (see Section 5.3 for details).

In this paper, we demonstrate birefringence imaging with computational refocusing. We adopt the Fresnel-diffraction-model-based computational refocusing [23] in Jones-matrix-based PS-OCT (JM-OCT) [19,34,35]. The computational refocusing processing not only refocuses the four individual polarization channels of the JM-OCT, but also preserves the phase consistency across the polarization channels. We validate this method by measuring a plastic foam phantom, postmortem zebrafish sample, and *ex vivo* porcine muscle samples. In addition, we present a detailed investigation of polarization artifacts caused by defocusing and their mitigation through computational refocusing.

## 2. Principle and core methods

### 2.1 Jones matrix optical coherence tomography

Jones matrix-based PS-OCT (JM-OCT) [19,34,35] was used for this research. JM-OCT provides four complex OCT images from a single scan, which are the entries of the “cumulative measured Jones matrix” ( $J_{m}$ ),

(1) $$J_{m}(x,y,z)=J_{out}J_{s}(x,y,z)J_{in},$$

where x and y are two transversal positions, and z is the depth position. $J_{out}$ and $J_{in}$ are the Jones matrices of input and output paths, respectively. $J_{s}(x,y,z)$ is the round trip Jones matrix of a sample.

A local Jones matrix corresponding to a depth region of [ $z_{1},z_{2}$ ] is defined as

(2) $$J_{l}(x,y,z_{1},z_{2})=J_{m}(x,y,z_{2})J_{m}^{-1}(x,y,z_{1})=J_{out}J_{s}(x,y,z_{2})J_{s}^{-1}(x,y,z_{1}){J_{out}}^{-1},$$

where $z_{2}>z_{1}$ . Since this local Jones matrix is a similar matrix to the sample’s local Jones matrix, $J_{s}(x,y,z_{2})J_{s}^{-1}(x,y,z_{1})$ , the eigenvalues of $J_{l}(x,y,z1,z2)$ and $J_{s}(x,y,z_{2})J_{s}^{-1}(x,y,z_{1})$ are identical, and hence the phase retardation of $J_{l}(x,y,z1,z2)$ is identical to that resulting from the local depth region in the sample. This phase retardation at the local depth region is denoted as local phase retardation.

The birefringence is proportional to the local phase retardation as

(3) $$b(z_{1},z_{2})=\delta (z_{1},z_{2})/2k_{0}Z_{d},$$

where $b(z_{1},z_{2})$ is the birefringence measured at [ $z_{1},z_{2}$ ], $\delta (z_{1},z_{2})$ is the local phase retardation, $k_{0}$ is the wave number corresponding to the center wavelength of the probe beam, and $Z_{d}=z_{2}-z_{1}$ , i.e., the depth size of the local region.

In this particular study, a swept-source JM-OCT with a 1.31- μm probing wavelength was used. The system is similar to that described in Ref. [35], but uses the k-clock-frequency-doubling mechanism described in Ref. [36]. In short, the light source is a wavelength sweeping laser (AXP50124-8, Axsun Technologies, MA) with a center wavelength of 1.31 μm and a sweeping rate of 50 kHz. The objective (LSM03, Thorlabs, NJ) has an effective focal length of 36 mm. The system provides lateral and depth resolutions in tissue of 18 μm and 14 μm , respectively. The depth-pixel separation in tissue is 7.24 μm . The probe arm has a passive polarization delay module (DE-G043-13, Optohub Co., Ltd, Saitama, Japan) that multiplexes two incident polarization states of the probe beam at two depths in the OCT image. The back-scattered signal from the sample is sent to a polarization diversity detection module (DE-G036-13, Optohub) where a reference beam interferes with it. Two interference signals of two output polarizations are then independently detected. These two signals are processed to yield OCT signals, as detailed in Ref. [35].

Because of the multiplexing of the incident polarization states and the polarization diversity detection, this system provides four complex OCT signals from a single scan. These four signals are denoted as four polarization channels, and they correspond to each entry of the cumulative measured Jones matrix [Eq. (1)].

Further details and specification of the system are described in our previous publications [35,36].

### 2.2 Computational refocusing

Volumetric computational refocusing based on a Fresnel-diffraction-model [23] was applied to each of the four OCT signals corresponding to the four polarization channels. Assuming an *en face* complex OCT is extracted from one of the four polarization channels at a depth where the defocusing distance is $z_{d}$ , the refocusing is performed by applying a phase-only spatial frequency filter,

(4) $$H^{-1}(f_{x},f_{y};z_{d})=\exp\left[-i\pi \lambda_{c}z_{d}\left(f_{x}^{2}+f_{y}^{2}\right)\right],$$

to the spatial frequency spectrum of the *en face* OCT, where $f_{x}$ and $f_{y}$ are the spatial frequencies corresponding to the lateral positions x and y , and $\lambda_{c}=2\pi /k_{0}$ is the center wavelength of the probe beam.

By assuming the depth position of the *en face* plane as z and expressing the OCT signal as S(x,y,z) , the refocusing operation is written as

(5) $$S^{\prime }(x,y,z)=F^{-1}\left\{F[S(x,y,z)]H^{-1}\left[\left(f_{x},f_{y};z_{d}\right)\right]\right\}$$

where $S^{\prime }(x,y,z)$ is the refocused *en face* OCT signal, and F[] and $F^{-1}[\;]$ denote transversal 2-D Fourier transform and inverse transform, respectively. In the numerical implementation, we additionally applied the aliasing-noise-removal method described in Section 5.2.

In practice, the defocusing amount $z_{d}$ is obtained from the measured OCT signal according to the following three steps. The first step is bulk-phase-error correction. In this step, a bulk phase error estimation based on a smart-integration-path [37] is used. This estimation is performed with a single polarization channel, and the same estimated bulk-phase-error is used to correct the bulk-phase errors of all four channels.

The second step is to estimate the defocusing amount at each depth $z_{d}(z)$ . In this step, $z_{d}$ is estimated at each depth in a depth-region of 50 pixels with sufficient OCT signal strength. This estimation is performed to minimize the information entropy of the linear intensity *en face* OCT image. This process is implemented with the Broyden-Fletcher-Goldfarb-Shanno algorithm [38] using the optimize.minimize function in the SciPy Python library. In our specific implementation, the accuracy of the optimization was set to 1 nm.

In the third step, the estimations of $z_{d}(z)$ in second step are fitted according to a linear function of z . Finally, the *en face* OCT signals at all depths are refocused again using the linear-fitted $z_{d}(z)$ .

After refocusing a polarization channel, the other three polarization channels are also refocused. For this refocusing, the linear-fitted $z_{d}$ obtained from the first channel is used.

Since the bulk-phase-error correction and the refocusing are performed with identical estimations of the bulk-phase-error and defocus, the phases among the four polarization channels are kept consistent during these processes.

### 2.3 Multi-contrast image formation

#### 2.3.1 Computation of OCT intensity, birefringence, and DOPU

OCT intensity, birefringence (local phase retardation), and DOPU images are then generated from the refocused Jones matrix tomography. The intensity OCT is the average of the four intensity OCT images corresponding to the four polarization channels.

The birefringence is computed from the local Jones matrix [Eq. (2)]. In our particular implementation, the separation of $z_{1}$ and $z_{2}$ , i.e., $Z_{d}$ , was set to 8 pixels (57.9 μm in tissue). In addition, a maximum *a-posteriori* (MAP) estimator was used to obtain the maximum likelihood estimation of birefringence [39,40].

DOPU [17] is a quantity associated with spatial randomness of the polarization , and is known to be sensitive to melanin [41–44]. In our particular implementation, DOPU was computed with Makita’s noise correction [45] from the Stokes vectors of two orthogonal input states using a spatial kernel of 3 × 3 pixels. Here the Stokes parameters of two input states were averaged, and then the norm of the averaged Stokes parameters were intensity-weighted-summed to compute the DOPU. See Eq. (3) in Ref. [45] for details.

#### 2.3.2 Pseudo-color image formation

For better visualization of the multi-contrast images, both birefringence and DOPU are composed with intensity, and pseudo-color multi-contrast images are created. The pseudo-color birefringence image is formed using the birefringence as its hue and the OCT intensity as the brightness. The saturation is defined by the estimation reliability of the birefringence [39,40]. Details on the image formation can be found in Section 3.4 of Ref. [40]

For the DOPU image, the first DOPU values are mapped using a rainbow color map, then the colored DOPU image is converted to hue-saturation-lightness color space. The saturation value is then replaced with the log-scale OCT intensity [46]. In this image, high DOPU is shown as red, low DOPU as yellow, and low-intensity regions as gray.

## 3. Samples and protocols of validation measurements

To demonstrate the implementation of computational refocusing in PS-OCT, two kinds of samples were examined, a plastic phantom and biological samples. Details of the measurement procedures are as follows.

### 3.1 Plastic phantom measurement

**Fig. 1.**
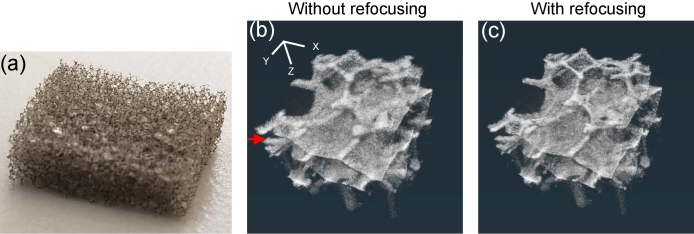
(a) The plastic foam used to fabricate the phantom. The plastic foam was embedded in silicone rubber. (b) and (c) are 3-D reconstructions of OCT intensity without (b) and with (c) computational refocusing. The red arrow indicates the depth location of Fig. 2. The 3-D scale bar in (b) denotes 500 μm .

A phantom consisting of a piece of polyethylene foam embedded in silicone rubber was imaged. The plastic foam measured had a size of approximately 1 cm × 1 cm with a thickness of around 2 mm, and a three-dimensional (3-D) polygonal structure [Fig. 1(a)]. Its birefringence characteristics are shown in the results (Section 4.1). Thirty-two mg of titanium dioxide micro-particles (TI-24-20-0110, Rare Metallic, Japan) were mixed with the non-birefringent silicone rubber (a mixture of 20 mL of 7600A and 7600B ELASTOSIL, Wacker Chemie AG, Germany) to enhance scattering. After embedding the foam in the silicone rubber, we let it rest to cure at room temperature for 24 hours.

### 3.2 Postmortem zebrafish and *ex vivo* porcine muscle measurement

In addition to the phantom, we also measured two biological samples. One was a postmortem adult wild-type zebrafish fixed with 4% paraformaldehyde. It was placed in a lateral posture in a petri dish for measurement. A piece of black tape was stuck to the bottom surface of the petri dish to prevent specular reflection of the probe beam.

The other biological sample was an *ex vivo* dissected porcine triceps brachii muscle. A slice of porcine muscle with a few millimeters thickness was cut along the muscle fiber. The sample was placed in a petri dish and immersed in saline solution.

These two biological samples were considered to contain birefringent components such as muscle fibers and collagen.

### 3.3 Measurement protocol

The samples were placed on a linear translation stage and measured by the JM-OCT system described in Section 2.1. To enhance light acquisition from the deep region, the focus of the probing beam was placed beneath the surface of the sample during the measurements.

The transversal scanning ranges were 3 mm × 3 mm for the phantom and 2 mm × 2 mm for the zebrafish and porcine muscles. All of the volumetric measurements consisted of 512 × 512 A-lines, which gave an isotropic lateral pixel separation of 5.86 μm for the phantom measurement and 3.90 μm for the zebrafish and porcine muscle. The volumetric acquisition time was 6.55 s.

## 4. Results

### 4.1 Plastic phantom

Figures 1(b) and (c) show the 3-D reconstructions of the log-scaled intensity image of the phantom, where Fig. 1(b) and (c) are the reconstructions without and with applying refocusing, respectively. Comparison of Fig. 1(b) and (c) reveals that the plastic walls are obviously thinner in the image with refocusing.

**Fig. 2.**
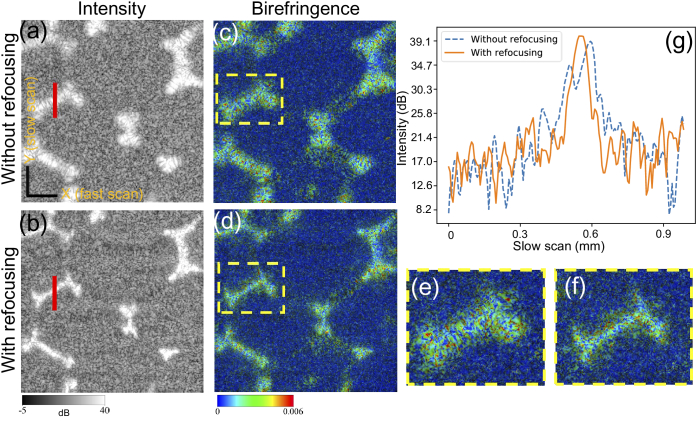
Intensity *en face* images without (a), and with (b) computational refocusing. (c) and (d) are the corresponding birefringence images, and (e) and (f) are magnified images of the yellow-box regions in (c) and (d), respectively. Scale bar denotes 500 μm . (g) Intensity line profiles at the red line in (a) and (b), where blue and orange curves denote the profiles without and with refocusing, respectively. Evident improvements in resolution following computational refocusing can be observed in the images and the plot.

Figures. 2(a)-(f) show *en face* intensity and birefringence images at the depth indicated by the red arrow in Fig. 1(b). The plastic walls appear broadened in the intensity OCT of Fig. 2(a) because of defocusing, while they appear sharp and thinner following computational refocusing [Fig. 2(b)]. Figure 2(g) presents line profiles at around the red lines in Fig. 2(a) and (b), to quantitatively evaluate the resolution improvement. They are the average of five adjacent A-scans along the horizontal (fast scan) direction. We measured the peak widths in Fig. 2(g) to represent the wall thickness. The peak widths of the profile with refocusing at -3 dB, -6 dB, and -10 dB of maximum were measured as 35 μm , 46 μm , and 76 μm , respectively, while the corresponding peak widths of the profile without refocusing were 41 μm , 58 μm , and 158 μm , respectively.

Figures 2(c) and (d) show the corresponding birefringence images. It can be seen that the plastic walls exhibit high birefringence (light blue to green), whereas the surrounding silicone rubber has lower birefringence (blue). Magnified images of the yellow-box regions in Fig. 2(c) and (d) are shown in Fig. 2(e) and (f), respectively. The birefringence of the plastic walls appears with almost the same color (light blue to green), regardless of the refocusing. This may suggest that the phases across the polarization channels are still consistent after the computational refocusing.

### 4.2 Postmortem zebrafish imaging

**Fig. 3.**
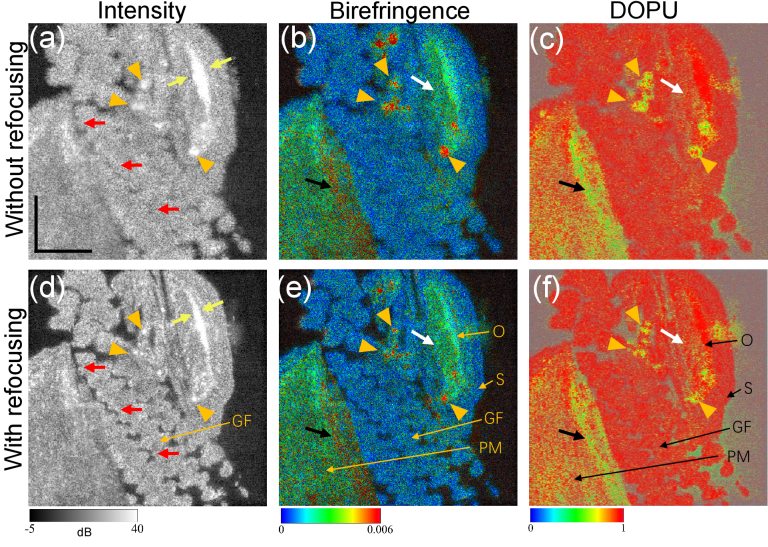
Zebrafish imaging results. (a)-(c) are the intensity, birefringence, and DOPU images without refocusing, respectively. (d)-(f) are the corresponding images with computational refocusing. Orange arrowheads indicate particles with strong reflection. Red arrows indicate the gill filament and yellow arrows the operculum. Black arrows indicate projection artifacts of birefringence and DOPU caused by structure superior to the imaged slice. White arrows indicate areas adjacent to the operculum, which might be operculum musculature or adductor mandibulae. Abbreviations: O, operculum; S, skin; GF, gill filament; PM, pectoral fin muscle. Scale bar denotes 500 μm .

Figure 3 shows multi-contrast images of the gill area of a previously healthy fixed zebrafish. The first row [Fig. 3(a)-(c)] shows images acquired before computational refocusing, and the second row [Fig. 3(d)-(f)] shows images after refocusing. After applying refocusing, it is possible to observe outlines and gaps between gill filaments (GF) in the OCT intensity images (red arrows), but these are not clearly visible without refocusing [Fig. 3(a) and(d)]. The yellow arrows indicate the operculum, the apparent cross sectional size of which is smaller after refocusing. In addition, several skin pigments with high scattering (orange arrowheads) are sharpened by the refocusing.

Figures 3(b) and (e) show the corresponding birefringence images. In these images, skin (S) and gill filament exhibit low birefringence (blue), whereas pectoral fin muscle (PM) exhibit relatively higher birefringence (green). The area being adjacent to the operculum also shows high birefringence, as the white arrows indicate. It is likely to be operculum musculature or part of the adductor mandibulae [47]. This area may contain muscle fiber or collagen which are birefringent. Operculum also shows high birefringence because it is collagen-rich [48].

In the DOPU images [Fig. 3(c) and (f)], S and GF show a homogeneous high DOPU appearance, while the muscle tissues including PM exhibit lower DOPU than S and GF, and the values are spatially inhomogeneous. Operculum exhibits high DOPU and high birefringence. It should be noted that the high birefringence and low DOPU stripe [black arrows in Fig. 3(b), (c), (e), and (f)] are projection artifacts from a superior layer.

These results suggest that the intensity, birefringence, and DOPU images can all be sharpened by computational refocusing.

### 4.3 Porcine muscle imaging

**Fig. 4.**
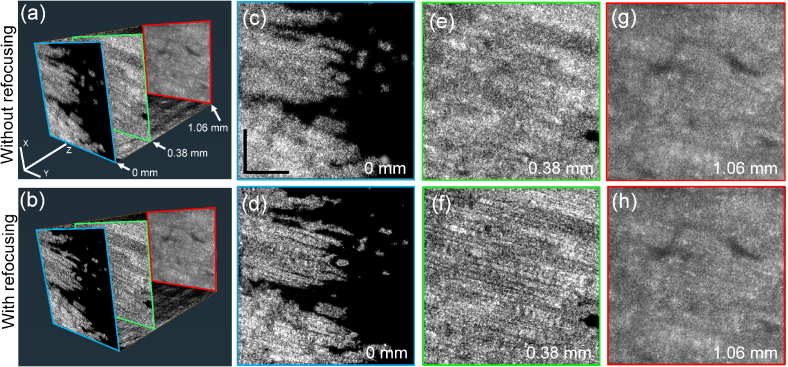
Original (first row) and computationally refocused (second row) OCT volumes of the porcine muscle sample. (a) and (b) 3-D reconstructed cut-away volumes. (c)-(h) are *en face* slices extracted from depths of 0-, 0.38-, and 1.06- mm from the surface. Scale bars in (a) and (c) denote 500 μm .

Figure 4 shows intensity OCT’s 3-D cut-aways and *en face* slices of a porcine muscle at 0-, 0.38-, and 1.06-mm depths, where 0-mm depth corresponds to the sample surface and the focus is located at around 1-mm depth. The first and second rows show images without and with refocusing, respectively. In the deep region (1.06-mm), the resolution improvement obtained with the refocusing was only moderate because it is close to the focus depth. However, at superficial depths (0-mm and 0.38-mm) the resolution is obviously improved by the refocusing, and fiber structures can be clearly visualized. This result demonstrates that a millimeter of imaging depth can be achieved, which makes the technique suitable for investigation of thick tissue samples.

**Fig. 5.**
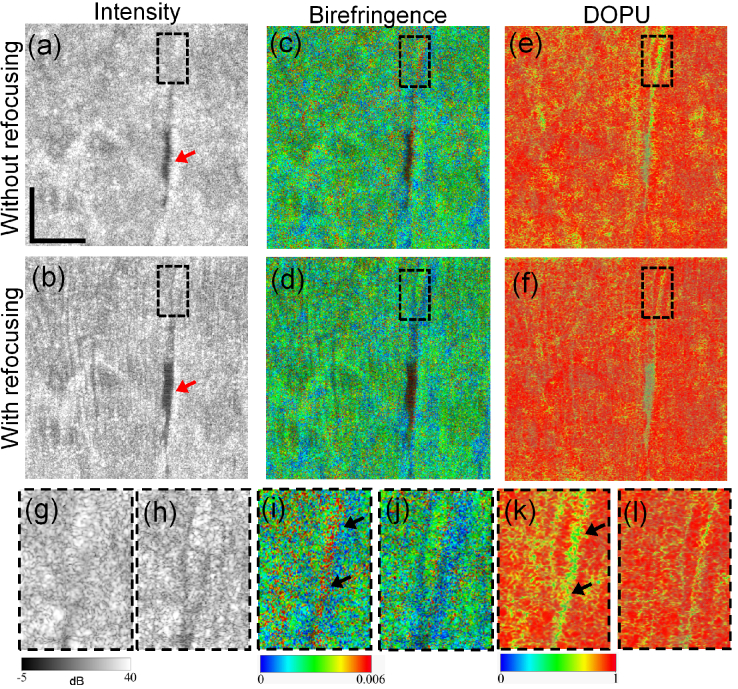
Original (first column) and computationally refocused (second column) *en face* porcine triceps brachii muscle images. The images are extracted from a depth away from the depth of focus. The first to third columns are the intensity, birefringence, and DOPU images, respectively. (g)-(l) are corresponding magnified images of the regions outlined by black-dashed boxes, where the left images are without refocusing and the right ones are with computational refocusing. Red arrows in (a) and (b) denote a hollow region, which exhibits evident sharpening following computational refocusing. Black arrowheads in (i) and (k) denote birefringence and DOPU artifacts. Scale bar denotes 500 μm .

*En face* intensity, birefringence, and DOPU images of a measured porcine muscle sample at a representative depth (0.28-mm from surface) are shown in Fig. 5 from left to right, respectively. The first row [Fig. 5(a), (c), and (e)] images are without refocusing, while the second row [Fig. 5(b), (d) and (f)] images are with refocusing. The third row shows magnified images at the regions indicated by the boxes. The left and right images of each contrast are without and with refocusing, respectively.

In the refocused intensity OCT image (the first column), vertically aligned fibrious structures are visible with clear boundaries [Fig. 5(b) and (h)]. However, they are not recognizable without refocusing [Fig. 5(a) and (g)]. A hollow region is visible at the center (red arrow), the edge of which also became sharp after refocusing.

The birefringence images [Fig. 5(c), (d), (i), and (j)] show a spatially inhomogeneous appearance. The fibers appear as high birefringence (green), while some of the surrounding connective tissues exhibit low birefringence (blue). This result is consistent with the knowledge that muscle fiber consists of oriented myofibrils, and hence is considered to be birefringent.

The DOPU images [Fig. 5(e), (f), (k), and (l)] show overall low and inhomogeneous DOPU without refocusing [Fig. 5(e) and (k)]. The refocusing made the low DOPU (yellow) regions appear sharp, and they are oriented along the muscle fiber. The other regions became homogeneous and show high DOPU (red).

We note that the refocusing seems to narrow down the high birefringence and low DOPU regions [Fig. 5(i)-(l)]. It suggests that defocusing tends to increase the birefringence of low birefringence regions, but not to decrease the birefringence of high birefringence regions. This finding is consistent with that reported by South *et al.* in respect to cumulative phase retardation [31]. Similarly, defocusing tends to decrease the DOPU of high DOPU regions, but not to increase the DOPU of low value regions. This effect is further investigated in Section 5.

## 5. Discussion

### 5.1 Artifacts in birefringence and DOPU

#### 5.1.1 Defocus-induced polarization artifacts

We here further investigate the alterations of the birefringence and DOPU obtained through computational refocusing, such as those shown in Section 4.3. To investigate whether the reduction in birefringence and increased DOPU obtained by computational refocusing are real effects or artifacts induced by the refocusing, another set of measurements were performed.

**Fig. 6.**
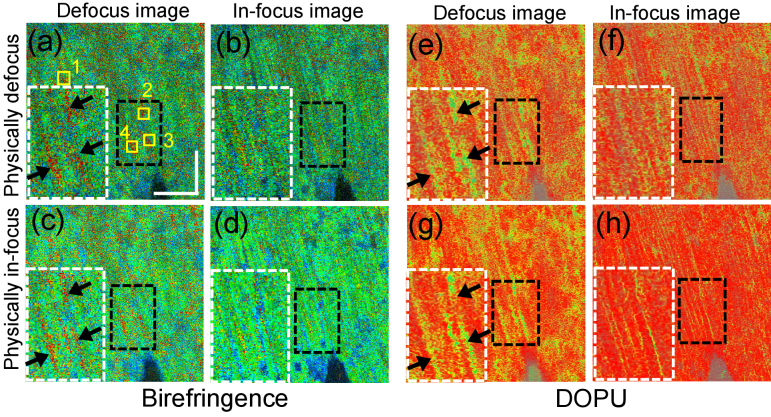
Comparisons of physically/computationally in-focus and physically/computationally defocused images. (a) and (e) are physically defocused images, and (b) and (f) are corresponding computationally refocused images. (d) and (h) are physically in-focus images, while (c) and (g) are computationally defocused versions of these images. The white-box insets are the magnified images of the black-box regions. The black arrows indicate the polarization artifacts. Yellow boxes denote four manually selected areas with a 15 × 15-pixel size where artifacts are observed. Scale bar denotes 500 μm .

A porcine triceps brachii muscle sample different to that imaged in Fig. 5 was measured using an identical protocol to that described in Section 3.2. The volumetric measurements were performed twice with different focus-positions. The first volume was acquired by placing the focus at 1-mm depth from the surface. The second volume was acquired by placing the focus near to the sample surface. *En face* images were extracted from these two volumes at the same depth close to the surface, as shown in Fig. 6. The raw images of the first volume were physically defocused [Fig. 6(a) and (e)] and computational refocusing was applied [Fig. 6(b) and (f)]. The raw images of the second volume are physically in-focus [Fig. 6(d) and (h)], and we then applied computational “defocusing” to them, as shown in Fig. 6(c) and (g), where the defocusing amount was the same as the refocusing amount used in Fig. 6(b) and (f). Therefore, the images in the first columns of each contrast in Fig. 6 are similarly defocused, while the second columns show similarly in-focus images. The white-box insets are magnified images of the regions in black boxes.

In the birefringence images, both the physically and computationally defocused images [Fig. 6(a) and (c), respectively] exhibit wide stripes with very high (red) birefringence (indicated by black arrows). These high birefringence stripes are not notable in either the computationally refocused or physically in-focus images [Fig. 6(b) and (d), respectively].

**Fig. 7.**
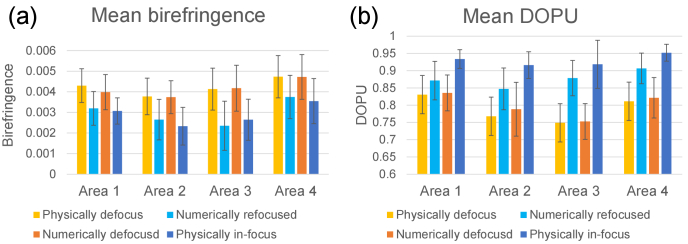
Mean birefringence (a) and DOPU (b) at four regions in Fig. 6 (yellow boxes). The mean values were computed as the mean of the mean values of each region. Bars indicate the standard deviations among the four means of the four regions.

To investigate this birefringence alteration in a more quantitative manner, the mean birefringence of small regions [yellow boxes in Fig. 6(a)] was computed for all four images. Each small region extends for 15 × 15 pixels (58.6 μm × 58.6 μm ). The mean birefringence values are plotted in Fig. 7(a), where the mean values were computed as the mean of the mean values of each region. Since all regions have the same size, this final mean is equivalent to the mean of all pixels for the four regions. The error bar shows the standard deviations of the four means of the four regions. Paired t-tests were performed to compare the mean birefringence over all four regions. Here the scipy.stats.ttest_rel function of SciPy 1.2.1 was used for the t-tests.

The computationally refocused image [Fig. 6(b)] shows statistically significant lower birefringence than the original physically defocused image [Fig. 6(a)] (p = 0.0061). Similarly, the physically in-focus image [Fig. 6(d)] shows significantly lower birefringence than its computationally defocused image [Fig. 6(c)] (p = 0.0027). However, there is not a significant difference between the two defocused images, nor between the two in-focus images (p = 0.4018, and 0.5566, respectively).

By regarding the physically in-focus birefringence image as a reference standard, the appearances of high birefringence in the physically and computationally defocused images can be considered to be artifacts, which can be suppressed by computational refocusing.

A similar investigation was performed for DOPU. In the *en face* images [Fig. 6(e)-(h)], low DOPU (yellow) stripes are reduced in size in both the computationally refocused image [Fig. 6(f)] and the physically in-focus image [Fig. 6(h)] in comparison with their defocused counterparts.

The mean DOPU values of the same region considered in the birefringence analysis are shown in Fig. 7(b). The computationally refocused DOPU image [Fig. 6(f)] shows a significantly higher mean DOPU value than its original physically defocused image [Fig. 6(e)] (p = 0.0184, paired t-test). Similarly, the physically in-focus image [Fig. 6(h)] exhibits a significantly higher mean DOPU value than its computationally defocused image [Fig. 6(g)] (p = 0.0025, paired t-test). On the other hand, no significant differences were found between physically and computationally defocused images [Fig. 6(e) and (g), p = 0.0813, paired t-test]. It should be noted that physically and computationally in-focus images [Fig. 6(f) and (h)] show moderate but significant differences (p = 0.0041, paired t-test) in image regions where the physically in-focus image shows higher mean DOPU than the computationally refocused image.

By regarding the physically in-focus DOPU image as a reference standard, the low DOPU appearances in the physically and computationally defocused images can be considered as artifacts. This artifact can be mitigated by computational refocusing, but this mitigation is not as good as it was with birefringence.

The moderate difference between physically and computationally in-focus images, i.e., the imperfection of the DOPU artifact removal by refocusing, could be partially explained by the imperfection of the computational refocusing. For example, in the Fresnel-diffraction-model-based refocusing, low and high order aberrations are known to interact at the spatial frequency spectrum of an OCT signal [49]. Therefore, the quadrature-phase spatial-frequency filter [Eq. (4)] cannot perfectly correct defocus if high-order aberration exists. This may result in imperfection in the artifact correction of DOPU.

#### 5.1.2 Dependency of polarization artifacts on the defocusing amount

The dependency of the polarization artifacts on the amount of defocusing was further investigated. We computationally applied different amounts of defocusing to the *en face* images shown in Fig. 3 and 5, and the physically in-focus image of Fig. 6. For each defocusing amount, the mean birefringence and mean DOPU values over the *en face* field were computed. In this example, the defocusing amount spanned from -0.8 to 0.8 mm, with a step of 0.1 mm, and low signal intensity regions that had a signal intensity lower than +5 dB of the noise floor were excluded from the mean computation.

**Fig. 8.**
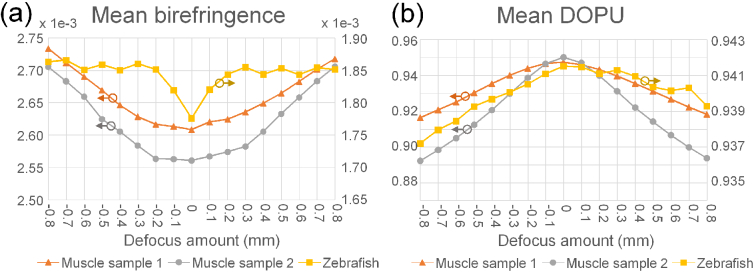
The dependency of the mean birefringence (a) and DOPU (b) on the amount of defocusing. Vertical axes indicate the mean birefringence (a) and DOPU (b) over the whole *en face* field except for low-intensity pixels. Each plot corresponds to different samples, with the orange rectangles, gray circles, and yellow squares denoting the first and second porcine muscle samples and zebrafish, respectively. As the absolute defocus increases, the mean birefringence increases and the mean DOPU decreases.

Figures 8(a) and (b) show the mean birefringence and DOPU values, respectively, as functions of the amount of defocusing. Red-triangles, gray-circles, and yellow-squares correspond to the data of Fig. 3 (zebrafish), Fig. 5 (the first porcine muscle), and Fig. 6 (physically in-focus data of the figure, to be denoted as the second porcine muscle in this section), respectively. The horizontal axis is the additional defocusing amount added after refocusing, where 0 represents the computationally in-focused image. Note that, since the original images were defocused, the zero-defocus position does not coincide with the physical in-focus position. The vertical axes denote the mean birefringence and mean DOPU values of the muscle samples (left) and zebrafish (right).

For porcine muscle tissues, it was found that the mean birefringence values monotonically increased as the defocus increased. Although it was not perfectly monotonic, a similar tendency was found with the zebrafish. DOPU monotonically decreased in response to increasing defocus for all of the samples, except for a minor fluctuation with the zebrafish. These findings suggest that the polarization artifacts become more significant as defocus increases.

### 5.2 Aliasing artifacts

**Fig. 9.**
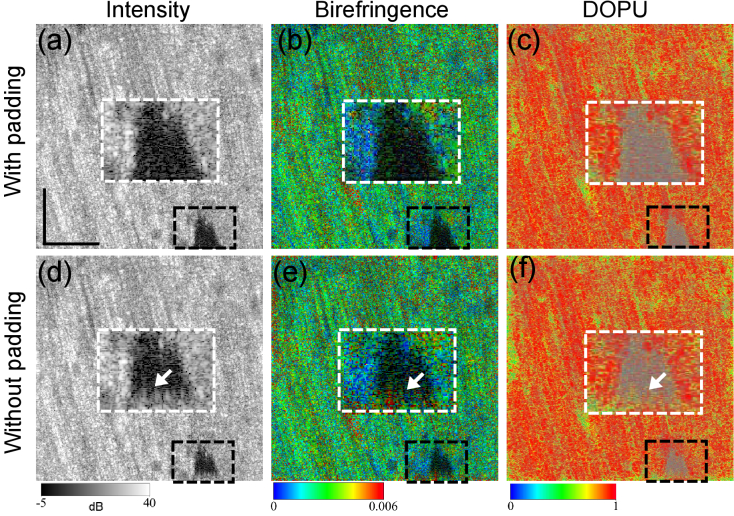
Artifacts that appear at the periphery of the *en face* field. The raw data are identical to what appear in Fig. 6. The first-to-third columns show the computationally refocused *en face* intensity, birefringence, and DOPU images, respectively. For (a)-(c), zero-fields were padded to the periphery of the image before the first Fourier transform of the computational refocusing process. On the other hand, zero-padding was not applied for (d)-(f). The white insets are magnifications of the black-box regions. Arrows in (d)-(f) indicate artifacts at the periphery. This artifact can be removed by zero-padding. The scale bar denotes 500 μm .

We frequently found an artifact in OCT and PS-OCT at the periphery of the *en face* field, as exemplified in Fig. 9. Here, the raw data are identical to what appear in Fig. 6. Figures 9(b) and (c) are identical to Fig. 6(b) and (f), respectively. Figures 9(d), (e), and (f) are the refocused results with the artifact (arrows). The white-box insets show magnified images of the black-box regions.

This artifact is the result of blurring of the sharp edge of the *en face* field caused by the computational refocusing. In detail, the computational refocusing sharpens the sample structure. However, the edge of the *en face* field, which can be considered as an artificial in-focus (sharp) structure in the image, is defocused (blurred) by the refocusing, and the blurred edge is then aliased into the other side of the field.

We avoided this artifact by extending the *en face* image field using zero-padding prior to the spatial frequency spectrum computation for the computational refocusing. In our particular implementation, we added numerical zero fields with a width of 50 pixels to all four sides of the *en face* field. After computational refocusing, the aliasing artifact appeared within this extended area, and was truncated afterwards.

### 5.3 Jones matrix OCT and circular-polarization light PS-OCT

Our method uses the JM-OCT principle. The advantage of JM-OCT over the CPL-PSOCT [32], which was used for the polarization sensitive (PS-) ISAM [31] and PS-CAO [33], is that it can provide birefringence, i.e., local phase retardation through local Jones matrix analysis [50]. The disadvantage of JM-OCT is that it requires phase information to compute both the cumulative and local phase retardations. Therefore, both the JM-OCT system and the refocusing algorithm should be phase-stable.

In contrast, the cumulative phase retardation measurement of CPL-PSOCT is phase insensitive. Therefore, its combination with computational refocusing, i.e., PS-ISAM [31] and PS-CAO [33], might be more robust than our computational-refocusing JM-OCT. The disadvantage of CPL-PSOCT is that the phase-insensitive method only provides the cumulative phase retardation and gives neither local phase retardation nor optic axis orientation. Note that the local phase retardation cannot be computed from the cumulative phase retardation (see Section 6.1 of Ref. [19], for example). Another limitation is that, even if we use the phase information, the phase retardation measurement can be highly sensitive to noise at a particular depth where the axis orientation of the tissue is in parallel or close to parallel to the polarization of the probe beam at that depth. In addition, the cumulative optic axis orientation cannot be determined when the cumulative phase retardation is an integer multiple of π [51].

In principle, CPL-PSOCT can also measure the local phase retardation if the sample does not have diattenuation, where the Jones matrices of the sample are unitary. In this case, a tomography of measured Jones matrices, which is equivalent to that measured by JM-OCT, can be reconstructed from the measured signals of CPL-PSOCT. The local Jones matrix can then be computed from this reconstructed measured Jones matrix, and hence the local phase retardation can be computed using the same algorithm as used for JM-OCT [52]. However, this method is phase sensitive, and therefore its robustness when combined with computational refocusing might be similar to our present method.

### 5.4 Limitations in *in vivo* measurement

PS-OCT and JM-OCT are known to be effective for ophthalmic diagnosis [19,53]. However, application of our method to *in vivo* eye measurement remains challenging, mainly because of sample motion and its resulting phase instability. Although our method uses a sophisticated phase stabilization algorithm [37], it is not sufficient for *in vivo* eye imaging. In addition, lateral motion also hampers the application of computational refocusing.

So far, computational aberration correction of *in vivo* human retinal imaging has been successfully demonstrated using short-acquisition-time point-scanning OCT [54,55]. The combination of PS-OCT and ultra-high-speed OCT may enable *in vivo* computational refocusing PS-OCT. In addition, Lissajous scan OCT has also been demonstrated for motion free *in vivo* retinal OCT [56] and OCTA imaging [57,58]. In the future, a combination of the Lissajous scan and the sophisticated phase stabilization algorithm may enable computational refocusing PS-OCT of *in vivo* retina with a standard-speed PS-OCT device.

### 5.5 Computational methods for improving microscope resolution

With the increasing computing power in recent years, several computational techniques to improve image resolution have become feasible. Neural-network-based superresolution and refocusing were demonstrated with linear and nonlinear microscopies [59,60]. Neural-network-based methods were also applied to improve the depth resolution of OCT [61,62].

More conventionally, physical-model-based methods have been demonstrated. For example, diffraction-computation-based refocusing is an essential part of digital holography and holographic microscopy [63–65]. Similar to digital holography, OCT also provides access to the complex optical field in the sample and its spatial frequency spectrum. Several computational refocusing techniques have been demonstrated by exploiting this property of OCT. It includes ISAM [24], Fresnel-diffraction-model-based computational refocusing [23], Gabor domain OCT [21], computational and digital adaptive optics [25,26,66].

Potentially, the computational refocusing and resolution improvements are applicable to PS-OCT. The present study and Ref. [31] (discussed in details in Section 5.3) are the examples. In addition, He *et al.* recently expounded the coupling effect of polarization and aberration [67]. Their theory, “vectorial adaptive optics,” can be adopted to further improve the computational refocusing and computational adaptive optics of PS-OCT.

### 5.6 Factors affecting the defocus estimation

#### 5.6.1 Impact of assumption used in defocus estimation

**Fig. 10.**
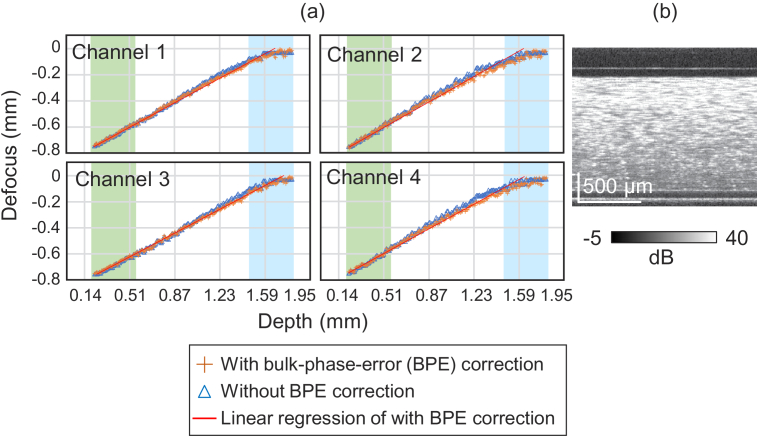
Defocus estimates are plotted against the sample depth. (a) Four plots represent four polarization channels, where each column and row correspond to each input and output polarizations, respectively. The defocus estimates were performed independently to each polarization channel. Orange crosses and blue triangles indicate the results with and without bulk-phase error correction, respectively. The red line is a linear regression line obtained from the data at the green region. The blue region indicates the region within the depth-of-focus (DOF). The data with bulk-phase error correction well agree with the regression line outside of DOF, while systemic departure can be found within the DOF as expected. (b) shows a representative cross-sectional OCT of the scattering phantom without refocusing.

In the present method, the defocus at each depth was estimated by optimizing the defocus parameter in the phase-only spatial frequency deconvolution filter [Eq. (4)] as it maximizes the sharpness of the image, i.e., minimizes the information entropy. During the derivation of Eq. (4) (or Eq. (12) in the original literature [23]), an assumption, $z_{0}\gg DOF/2$ , was used, where DOF is depth-of-focus. And hence, accurate defocus estimation is not guaranteed within the DOF. This issue is highlighted in Fig. 10, where the estimated defocus at each polarization channel was plotted against the sample depth [Fig. 10(a)]. The estimated defocus at around the focus (blue region) is not linear to the sample depth. Here the sample is a scattering phantom [Fig. 10(b), cross-sectional OCT], and the defocus estimation was performed independently for four polarization channels. Orange crosses and blue triangles indicate the results with and without bulk-phase error correction, respectively. The details of the phantom and the measurements are described later in this section.

For the depth region near or within the DOF, the defocus amount was estimated by extrapolation. The region used for the extrapolation is highlighted by green in Fig. 10(a). This estimation might be reasonable because of two reasons. At first, the defocus is small enough for the depth region within DOF. And hence, precise defocus correction is not necessary. Second, the deconvolution filter [Eq. (4)] becomes a very weak quadratic phase function in this region. So, the effect of estimation error is negligible.

The details of the phantom and the measurement of Fig. 10 are following. The phantom is a non-birefringent scattering phantom, which is a mixture of micro-particles with a 10- μm diameter (72968-10ML-F, Sigma-Aldrich) and ultrasound gel (Pro Jelly, Jex, Japan). A cover glass was placed on top and was tilted to prevent specular reflection. An OCT volume consists of 512 × 512 A-scans, which covers a transversal scanning range of 1.5 mm × 1.5 mm, and hence a lateral pixel separation is 2.93 μm . The focus was placed roughly at 1.6-mm depth from the surface.

#### 5.6.2 Impact of bulk phase correction in defocus estimation

To evaluate the impact of bulk-phase error correction on defocus estimation, we estimated the defocus of the scattering phantom image with and without the bulk-phase error correction as shown in Fig. 10. The details of the phantom and the measurement have been described in Section 5.6.1. The defocus estimates are linear to the depth location with the bulk-phase error correction (orange crosses) as expected. One the other hand, those without bulk-phase error correction (blue triangles) are not well linear at around 1.2-mm depth. Note that the departure from the linear line at around the focus has been discussed in Section 5.6.1.

### 5.7 Future work

#### 5.7.1 Possible effect from polarization to refocusing

Recently, He *et al.* have been intensively exploring the coupling of aberration and polarization [67,68]. Its theory, “vectorial adaptive optics” implies that not only the phase but also the polarization may affect the physical focus. And hence, the both and polarization also affect the computational refocusing. Investigation of this issue and extending computational refocusing PS-OCT with the vectorial adaptive optics theory might be worth exploring in the future.

#### 5.7.2 Validation with standardized/calibrated birefringent phantom

In this study, we used a self-made plastic phantom and biological samples to validate the refocusing performance. However, these samples do not have known and calibrated birefringence. In the future, introduction of calibrated and standardized birefringent phantoms [69–71] will be helpful to further generalize our conclusion.

#### 5.7.3 Physical mechanism of the polarization artifact

In this paper, we found that the defocus artifactually increases the birefringence and decreases DOPU. This birefringence artifact can be caused by interaction between the defocused OCT signal and birefringence-reconstruction algorithm [19,34,50] or its employing maximum a-posteriori birefringence estimator [39,40]. Note that the defocus effect is not considered in the estimator’s theory. Although Ruiz-Lopera *et al.* showed a similar artifact tendency [72], its physical principle is still not well investigated.

The low DOPU artifact may relate to its kernel-based computation. If the kernel covered inhomogeneous regions, the computed DOPU would decrease. Similarly, the more defocus exits, the point spread function would be more widened. It also extends the spatial region used to compute DOPU. It may artifactually decrease DOPU.

One possible investigation method of these issues is a numerical simulation. In this simulation, numerical tissue structures with wavelength-scale microstructures and a-few-micrometer-to-millimeter-scale macroscopic structures are considered, and PS-OCT signals with arbitrary defocus from the known numerical tissue structures are simulated. For example, the authors have numerically investigated the polarization property of OCT signals with simple micro-structured samples [73]. Although this previous study did not take the spatial extent of the probe beam into account, future extension of such methods can help investigate this issue.

Another possible factor affecting the polarization artifact is multiple scattering. For scattering samples, the multiple scattering signals also artifactually affect the birefringence and DOPU measurements. And the behavior of multiple scattering could be affected by defocus and aberrations. In the future, it is worth investigating the effect of multiple-scattering-photon-rejection methods [74–77] on the polarization artifacts.

## 6. Conclusion

In this study, we demonstrated computational refocusing using Jones matrix-based PS-OCT. The method was validated by measuring a plastic phantom, a postmortem zebrafish, and *ex vivo* porcine muscle samples. The results showed that the lateral resolutions at depths outside the depth-of-focus were significantly improved for all of the OCT, birefringence, and DOPU measurements. We also found that defocus increases the measured birefringence value and decreases the measured DOPU value. Although this artifact must hamper the quantitative polarization measurement, it was also found that this artifact can be significantly mitigated by computational refocusing. Hence, computational refocusing is important not only for the observation of fine structures, but also for quantitative birefringence and DOPU measurements.

## Data Availability

Data underlying the results presented in this paper are not publicly available at this time but may be obtained from the authors upon reasonable request.
